# Reduced Lower Body Muscular Strength and Endurance among Adult Survivors of Childhood Cancer

**DOI:** 10.1249/MSS.0000000000003593

**Published:** 2024-11-15

**Authors:** TOMÁŠ SLÁMA, CARINA NIGG, RETO D. KURMANN, GABRIELA M. KUSTER, NANA K. POKU, EVA SCHELER, CLAUDIA E. KUEHNI, NICOLAS X. VON DER WEID, CHRISTINA SCHINDERA

**Affiliations:** 1 Childhood Cancer Research Group, Institute of Social and Preventive Medicine, University of Bern, Bern, SWITZERLAND; 2 Graduate School for Cellular and Biomedical Sciences, University of Bern, Bern, SWITZERLAND; 3 Division of Cardiology, Heart Center, Luzerner Kantonsspital, Lucerne, SWITZERLAND; 4 Division of Cardiology, University Hospital Basel, Basel, SWITZERLAND; 5 Cardiology Division, University Hospital of Geneva, Geneva, SWITZERLAND; 6 Division of Cardiology, Department of Internal Medicine, Hospital of St. Gallen, SWITZERLAND; 7 Pediatric Oncology, Inselspital, Bern University Hospital, University of Bern, SWITZERLAND; 8 Division of Pediatric Oncology/Hematology, University Children's Hospital Basel, University of Basel, Basel, SWITZERLAND

**Keywords:** MUSCLE STRENGTH, ENDURANCE, OBESITY, CISPLATIN, CRANIAL RADIOTHERAPY, LONGITUDINAL STUDY

## Abstract

**Introduction:**

Impaired physical fitness is a possible late effect among adult survivors of childhood cancer (ASCC). Our study describes lower body muscular strength and endurance among ASCC using the 1-min sit-to-stand (1-min STS) test, compares them with the general population, identifies risk factors, and describes changes over time.

**Methods:**

In a prospective multicenter cohort study, we invited ASCC ≥18 yr of age at study who were diagnosed between ages 0 and 20 yr, treated in five pediatric oncology centers across Switzerland from 1976 to 2017, and survived ≥5 yr for a 1-min STS test. We collected information about lifestyle, medical history, and previous cancer treatment. Using population-based Swiss reference values, we calculated age- and sex-adjusted *z*-scores for 1-min STS performance and assessed the association between risk factors and 1-min STS test using multivariable linear regression. We fitted a multilevel linear model to describe the longitudinal course of 1-min STS performance.

**Results:**

We included 338 CCS of 1048 invited ASCC (participation rate 32%) with a median age at study of 34 yr (interquartile range, 26–41 yr). Compared with the general population, mean 1-min STS *z*-score was half a standard deviation lower (−0.52; 95% confidence interval (CI), −0.64 to −0.40). Obesity (*B* = −0.56; 95% CI, −0.97 to −0.16), cumulative cisplatin dose (*B* = −0.12; 95% CI, −0.21 to −0.02), and cumulative cranial radiotherapy dose (*B* = −0.10; 95% CI, −0.19 to −0.01) were associated with reduced 1-min STS performance. There was no change in 1-min STS *z*-scores over time (*B* = 0.02; 95% CI, −0.05 to 0.09).

**Conclusions:**

We found evidence for reduced lower body strength and endurance among ASCC, suggesting the need for counseling and effective training and rehabilitation programs for maintaining daily functioning, improving cardiovascular health, and reducing morbidity for ASCC.

Impaired physical fitness—with muscular strength and endurance as two core components (1)—is a possible late effect among adult survivors of childhood cancer (ASCC); it results in reduced mobility and walking efficiency, increased cardiometabolic risk, and early mortality (2). Impaired muscular endurance limits repeated functional movements, reducing the ability to engage in physical tasks necessary for everyday life as well as participation in physical activities and sport (2). Survivors of central nervous system tumors have been shown to be particularly affected by deficits in lower body strength and muscular endurance (3). Previous studies identified several treatment-related risk factors for lower body muscular strength and endurance, including asparaginase (4), vincristine (5), cranial radiotherapy (CRT) (4,6), and hematopoietic stem cell transplantation (7,8). Similar to the general population, lower body mass index (BMI) was associated with better muscular performance among survivors of acute lymphoblastic leukemia (ALL) (8). Evaluating lower body strength and endurance among survivors is crucial for assessing mobility and exercise capacity, which then serves as a foundation for advising effective training and rehabilitation strategies to promote muscular strength and endurance.

The sit-to-stand (STS) test has been used extensively and is reliable to assess lower body muscular strength and endurance in clinical settings (9,10), including among survivors of cancer (11,12). It assesses lower body muscles, especially quadriceps strength (13,14), aerobic and anaerobic capacity, and sensorimotor, and balance components (9,15). There are several variations of the STS test, such as five-repetition STS test, 30-s STS test, and 1-min STS test (16–18). Among deficits of lower body muscular strength and endurance of ASCC were described absent Achilles tendon reflexes (19), impaired knee extension strength (19,20), and a decreased ankle dorsiflexion range of motion (19,21). Because ASCC with reduced lower body strength and endurance more often report poor general health, functional impairments, and activity limitations (22), it is important to study this phenomenon to better understand the long-term impact of childhood cancer on survivors’ daily lives. Previous studies showed that lower body strength and endurance assessed with the Biodex III Myometer device are reduced among ASCC when compared with a matched control population (19,21). Although such devices allow very accurate assessment, they are not widely applicable in routine clinical care due to their high costs and logistical barriers (e.g., finding room for the device). Studies investigating cost-effective ways of lower body strength and endurance assessment among ASCC in routine clinical setting are lacking. Because of its minimal requirements on equipment, the 1-min STS test can be performed easily in any clinical setting. Furthermore, Swiss population-based reference values are available for 1-min STS performance among adults (23). With our study, we investigated lower body strength and endurance of ASCC using the cost- and resource-effective 1-min STS test within routine clinical follow-up care. We aimed to 1) describe lower body strength and endurance in a large cohort of ASCC with various childhood cancer diagnoses compared with the general population using the 1-min STS test, 2) identify lifestyle and treatment-related risk factors for a diminished 1-min STS performance, and 3) describe the development of 1-min STS performance in ASCC over a period of 2–5 yr.

## METHODS

### Study Design and Population

Our study is part of the Swiss CardioOnco study, which investigates cardiovascular health among ASCC in a routine care setup. The study protocol has been published (24). The CardioOnco study was initiated in 2016 as a single-center study at the University Hospital Inselspital in Bern, Switzerland. In 2021, we moved to a multicentric study setting also including University Hospital Basel, University Hospital of Geneva, Cantonal Hospital Lucerne, and Cantonal Hospital of St. Gallen. The study invites all ASCC diagnosed since 1976 treated at one of these five centers with any chemotherapy and/or heart-relevant radiotherapy to participate. At recruitment, ASCC had to be older than 18 yr, ≥5 yr since diagnosis, and registered in the Swiss Childhood Cancer Registry (ChCR). ChCR includes all individuals in Switzerland diagnosed before the age of 20 yr with any childhood cancer coded according to the International Classification of Childhood Cancer, Third Edition (25,26). We excluded survivors treated with surgery only and/or radiotherapy other than heart-relevant radiotherapy. Study teams in participating centers invited eligible survivors identified by the ChCR by post to visit the cardio-oncology clinic of the center where they were treated for childhood cancer previously. During clinical visits, treating physicians and study teams took medical histories and performed physical examinations, including the 1-min STS test. We excluded participants with leg prostheses and neurocognitive or visual impairments who were unable to correctly perform 1-min STS test. Because ASCC who received anthracyclines and/or heart-relevant radiotherapy are at increased risk for cardiovascular disease (27), they were invited to a follow-up visit 2–5 yr after the baseline assessment, depending on the cumulative dose of cardiotoxic treatment they received as recommended by international guidelines (28). Those who received a cumulative dose of anthracyclines of ≥250 mg·m^−2^, or cumulative dose of heart-relevant radiotherapy ≥35 Gy, or a combination of cumulative anthracycline dose <250 mg·m^−2^ and cumulative dose of heart-relevant radiotherapy ≥15 Gy were invited to a follow-up visit every 2 yr. Those who received a cumulative dose of heart-relevant radiotherapy ≥15 and <35 Gy, or a combination of cumulative anthracycline dose <250 mg·m^−2^ and cumulative dose of heart-relevant radiotherapy <15 Gy were invited to a follow-up visit every 5 yr. These intervals were shortened if clinically indicated by the treating cardiologist. We obtained written informed consent from all participants included in the study. Before the initiation of the study in additional centers, we prepared a standard operating procedure protocol describing the procedure of data acquisition and distributed it to new collaborators. The core team from the University of Bern was present in newly joining centers at the initiation of the data collection to oversee and further instruct local study teams to unify data collection mechanisms. We performed our study aligned with the principles of the Declaration of Helsinki. Ethical approval was granted by the Ethics Committee of Canton Bern (KEK-BE: 2017-01612).

### Population Characteristics

#### Demographic characteristics

When taking medical histories, we collected data on sex, age at study, marital status, number of children in the household, and employment status.

#### Lifestyle characteristics

During the visit, treating physicians asked survivors about their smoking status. A dedicated study nurse performed anthropometric measurements to obtain survivor’s BMI and waist–hip ratio. We measured hip circumference at the widest circumference over the buttocks and waist circumference at the midpoint between the lower margin of the lowest rib and the top of the iliac crest while participants stood with both feet tight together with a measuring tape (24). Devices used to assess weight and height were not identical across study centers. Sensitivity analysis of weight and height measurements across study centers showed a difference in weight measurements but no difference in height measurements among centers (Supplemental Tables 1A and 1B, Supplemental Digital Content, http://links.lww.com/MSS/D120). We calculated the BMI as the weight in kilograms divided by the square of the height in meters (29). We applied the World Health Organization cutoff points to classify BMI as underweight (<18.5 kg·m^−2^), normal weight (18.5–24.9 kg·m^−2^), overweight (25.0–29.9 kg·m^−2^), and obesity (≥30 kg·m^−2^) (29); we also classified abdominal obesity (waist-to-hip ratio of ≥0.90 in men and ≥0.85 in women) (30). From survivor’s medical history and medical records, we gathered data about dyslipidemia, hypertension, and diabetes mellitus.

#### Clinical characteristics

ChCR provided data on cancer diagnosis, age at cancer diagnosis, time since diagnosis, and relapse history. We asked survivors during clinical visits about second primary malignancies and validated with medical records. We collected information, including cumulative doses, from medical records on known and suspected cardiotoxic treatments, (31) including anthracyclines, cyclophosphamide, cisplatin, and heart-relevant radiotherapy. We calculated doxorubicin equivalent dose of different anthracyclines as suggested by the Children’s Oncology Group (28). We defined heart-relevant radiotherapy as any therapeutic exposure of the chest, abdomen, spine (thoracic or whole), and total body irradiation (TBI) (28). We further included risk factors for diminished muscle strength and endurance as described previously—asparaginase, vincristine, CRT, and stem cell transplantation (32). If a survivor received TBI, we added the dose to heart-relevant radiotherapy or CRT.

### One-Minute STS Test

We used the 1-min STS test because a Swiss population-based reference values exist (23) and previous studies showed a high degree of correlation between the 1-min STS test and other measures of physical functioning, such as 6-min walk test (*r* = 0.75, *P* ≤ 0.001) (33) or stair climbing (*r* = 0.68, *P* < 0.001) (34). Study nurses directed participants to complete sitting and standing positions on chairs of standard height without armrests (23). Participants kept their arms stationary or placed them on their hips; they were not allowed to use their arms during the test (23). At the start of the test, the study participants were instructed to sit in the middle of the chair with their back straight. Study participants were allowed to lean forward slightly when transitioning from sitting to standing. During the standing position, participants were required to straighten their torso completely upright. Study personnel directed and motivated participants to perform as many STS repetitions as possible within 1 min (23). We only counted completed STS repetitions—fully extended legs (standing) and participant buttocks touching chairs (sitting) (23).

### Statistical Analyses

First, we tested normality in the distribution of 1-min STS repetitions and found that skewness was 0.54 and kurtosis was 3.03. The distribution of 1-min STS repetitions can thus be considered normal (35). Second, we calculated *z*-scores for the 1-min STS test using sex- and age-stratified reference values (Supplemental Table 2, Supplemental Digital Content, http://links.lww.com/MSS/D120). To compare the *z*-score of our sample with the reference population, we used a one-sample *t*-test (statistical power = 100% based on a *post hoc* power analysis). Next, we fitted univariable linear regression models to identify lifestyle and clinical risk factors for a diminished 1-min STS *z*-score. For multivariable analysis (statistical power = 100% based on a *post hoc* power analysis), we included variables associated with 1-min STS performance in the unadjusted analysis at *P* < 0.1. We calculated *P* values using the Wald test. To analyze 1-min STS performance longitudinally, we included those survivors who completed the 1-min STS test in at least two consecutive clinic visits. These were ASCC with increased risk for cardiovascular disease. We fitted a multilevel linear model to assess change in performance of 1-min STS test over time. We nested time points within participants, which allowed us to consider all available time points for each participant and to model both fixed and random effects (36,37). We centered time on the baseline assessment. All *P* values are two-sided; we considered *P* < 0.05 statistically significant. We performed all analyses using Stata software, version 16.1 (StataCorp. 2019. Stata Statistical Software: Release 16. College Station, TX: StataCorp LLC.).

## RESULTS

### Characteristics of Study Population

**FIGURE 1:**
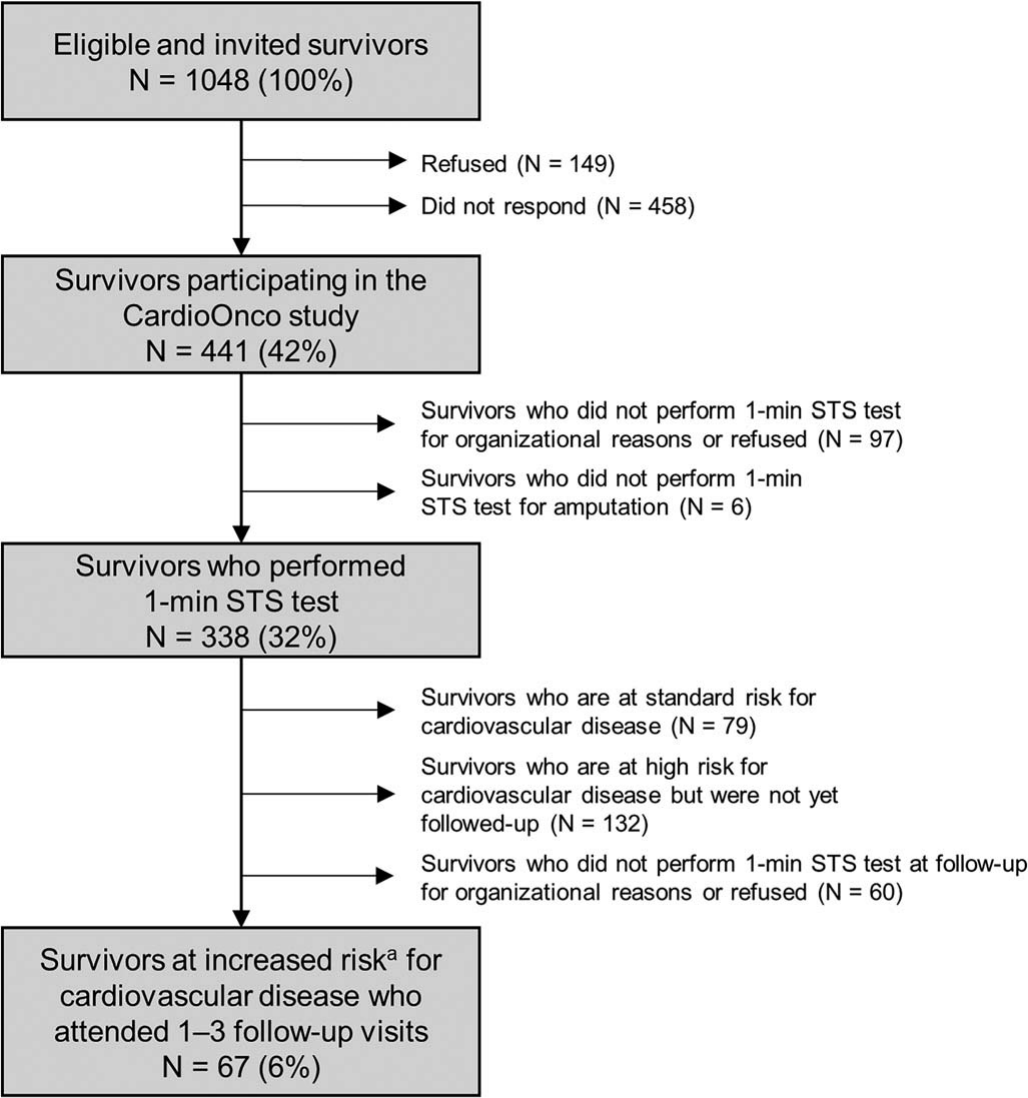
Flowchart of the study population. *N*, number. ^a^Defined as having received anthracyclines and/or heart-relevant radiotherapy (27).

Between January 2016 and November 2022, we invited 1048 eligible ASCC to join the CardioOnco study. Of those, 441 (42%) participated, 149 (14%) declined participation, and 458 (44%) did not respond. The test was not performed in 97 participants for organizational reasons (lack of time during clinical visits) or refusal, and in 6 because of an amputation, leaving 338 (77% of participants) who performed a 1-min STS test (Fig. 1). Median age at study was 34 yr (interquartile range (IQR), 26–41 yr) and at cancer diagnosis 6 yr (IQR, 3–12 yr; Tables 1A and 1B). Leukemia (42%) and lymphoma (22%) were the most frequent cancer diagnoses. According to BMI, we categorized 32% of participants with overweight and 10% with obesity. We present median cumulative doses of chemotherapy and radiotherapy in Table 1B.

**TABLE 1A:** Characteristics of study population.

	Total (*N* = 338), *n* (%)*^a^*	Male (*N* = 179), *n* (%)*^a^*	Female (*N* = 159), *n* (%)*^a^*
Demographic characteristics			
Age at study, median [IQR], yr	34 [26–41]	34 [27–42]	34 [24–40]
<30	121 (36)	59 (33)	62 (39)
30–39	121 (36)	66 (37)	55 (35)
≥40	96 (28)	54 (30)	42 (26)
Married, yes	122 (36)	66 (37)	56 (35)
Children, yes	103 (30)	53 (30)	50 (31)
Employment, yes	280 (83)	162 (91)	118 (74)
Lifestyle characteristics			
Smoking			
Never	221 (65)	114 (64)	107 (67)
Former	46 (14)	25 (14)	21 (13)
Current	71 (21)	40 (22)	31 (20)
BMI, median [IQR],*^b^* kg·m^−2^	24.0 [21.5–27.1]	24.7 [22.2–27.5]	23.2 [21.0–`26.6]
Underweight	16 (5)	4 (2)	12 (8)
Normal weight	180 (53)	91 (51)	89 (56)
Overweight	109 (32)	68 (38)	41 (26)
Obese	33 (10)	16 (9)	17 (11)
Waist–hip ratio*^c^*	0.9 [0.8–0.9]	0.9 [0.8–1.0]	0.8 [0.8–0.9]
No abdominal obesity	208 (62)	100 (56)	108 (68)
Abdominal obesity present	130 (38)	79 (44)	51 (32)
Dyslipidemia, yes*^d^*	23 (7)	15 (8)	8 (5)
Hypertension, yes*^d^*	36 (11)	18 (10)	18 (11)
Diabetes, yes*^d^*	9 (3)	4 (2)	5 (3)

*^a^* Column percentages. *^b^* BMI was classified as underweight (<18.5 kg·m^−2^), normal weight (≥18.5–<25 kg·m^−2^), overweight (≥25–<30 kg·m^−2^), and obese (≥30 kg·m^−2^) (29). *^c^* Abdominal obesity was defined according to WHO cutoff point as waist–hip ratio ≥0.90 in men and ≥0.85 in women (30). *^d^* As reported by survivors during standardized interview or found in their medical records. *N*, number.

**TABLE 1B:** Clinical characteristics of study population.

	Total (*N* = 338), *n* (%)^a^	Male (*N* = 182), *n* (%)*^a^*	Female (*N* = 162), *n* (%)*^a^*
Age at diagnosis, median [IQR], yr	6 [3–12]	6 [2–12]	6 [3–11]
Time since diagnosis, median [IQR], yr	27 [20–34]	29 [21–34]	26 [19–34]
ICCC-3 cancer diagnoses			
I Leukemias	141 (42)	62 (35)	79 (50)
II Lymphomas	74 (22)	48 (27)	26 (16)
III Central nervous system tumors	18 (5)	13 (7)	5 (3)
IV Neuroblastoma	11 (3)	8 (4)	3 (2)
V Retinoblastoma	3 (1)	2 (1)	1 (1)
VI Renal tumors	28 (8)	12 (7)	16 (10)
VII Hepatic tumors	4 (1)	3 (2)	1 (1)
VIII Malignant bone tumors	15 (4)	7 (4)	8 (5)
IX Soft tissue sarcomas	21 (6)	10 (6)	11 (7)
X Germ cell tumors	6 (2)	3 (2)	3 (2)
XI–XII Other tumors	17 (5)	11 (6)	6 (4)
*Total IV–XII*	105 (31)	56 (31)	49 (31)
Relapse, yes	41 (12)	23 (13)	18 (11)
Second primary neoplasia, yes	25 (7)	15 (8)	10 (6)
Hematopoietic stem cell transplantation, yes	26 (8)	16 (9)	10 (6)
Anthracyclines, yes	219 (65)	115 (64)	104 (65)
Cumulative anthracyclines, median [IQR],*^b^* mg·m^−2^	159 [120–250]	151 [120–233]	160 [122–260]
>0 and <250 mg·m^−2^	164 (49)	89 (50)	75 (47)
≥250 mg·m^−2^	55 (16)	26 (15)	29 (18)
Cyclophosphamide, yes	193 (57)	106 (59)	87 (55)
Cumulative cyclophosphamide, median [IQR], g·m^−2^	4 [3–6.3]	4 [3–5.6]	4.2 [2.6–6.8]
>0 and <4 g·m^−2^	90 (27)	54 (30)	36 (23)
≥4 g·m^−2^	103 (30)	52 (29)	51 (32)
Cisplatin, yes	31 (9)	21 (12)	10 (6)
Cumulative cisplatin, median [IQR], mg·m^−2^	385 [360–480]	390 [360–480]	383 [360–480]
>0 and <390 mg·m^−2^	16 (5)	10 (6)	6 (4)
≥390 mg·m^−2^	15 (4)	11 (6)	4 (3)
Asparaginase, yes	66 (20)	30 (17)	36 (23)
Vincristine, yes	226 (67)	109 (61)	117 (74)
Cranial RT, yes	66 (20)	38 (21)	28 (18)
Cumulative cranial RT, median [IQR],*^c^* Gy	24 [12–45]	24 [12–50]	24 [12–43]
>0 and <35 Gy	41 (12)	23 (13)	18 (11)
≥35 Gy	25 (7)	15 (8)	10 (6)
Heart-relevant RT, yes*^d^*	91 (27)	51 (28)	40 (25)
Cumulative heart-relevant RT, median [IQR],*^d^* Gy	24 [15–36]	26 [18–36]	22 [14–30]
>0 and <15 Gy	20 (6)	10 (6)	10 (6)
≥15 and <35 Gy	46 (17)	24 (13)	22 (13)
≥35 Gy	25 (7)	17 (10)	8 (5)

*^a^* Column percentages. *^b^* Doxorubicin-equivalent doses calculated according to the COG Guidelines Version 5.0 (28). *^c^* Including survivors who received TBI. *^d^* According to the COG Guidelines Version 5.0 (chest, abdomen, whole or thoracic spine, TBI) (28). *N*, number; RT, radiotherapy.

### Survivors’ 1-Min STS Performance and Comparison to the Reference Population

**FIGURE 2:**
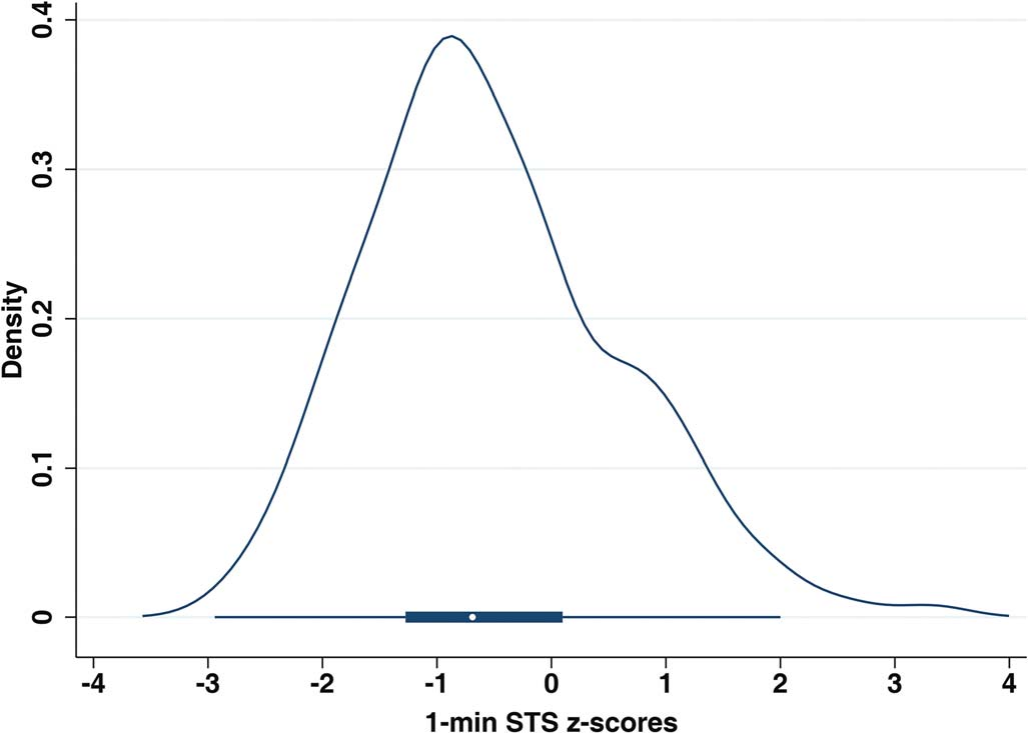
Distribution of 1-min STS *z*-scores in *N* = 338 ASCC who underwent baseline assessment on a half violin plot. The box plot below shows the median and IQR of the 1-min STS *z*-scores.

Median 1-min STS repetitions in our cohort were 39 (IQR, 32–48). Male participants performed a median of 41 (IQR, 32–50) STS repetitions, and females performed 38 (IQR, 32–45). Mean 1-min STS *z*-score of our cohort was −0.52 (95% confidence interval (CI), −0.64 to −0.40; *P* < 0.001; Fig. 2).

### Risk Factors for a Diminished 1-Min STS Performance among ASCC

We show univariable linear regression results in Supplemental Tables 3A–B (Supplemental Digital Content, http://links.lww.com/MSS/D120). For the reason of collinearity with treatment variables, we excluded cancer diagnosis in the final regression model. In multivariable regression, we found obesity (unstandardized coefficient (*B*) = −0.56; 95% CI, −0.97 to −0.16), cumulative cisplatin dose (*B* = −0.12; 95% CI, −0.21 to −0.02), and cumulative CRT dose (*B* = −0.10; 95% CI, −0.19 to −0.01) associated with diminished performance in 1-min STS test (Table 2). No other significant associations were found.

**TABLE 2:** Association between risk factors (BMI, cisplatin, CRT, and heart-relevant radiotherapy) and lower body muscular strength and endurance (1-min STS performance) among ASCC (*N* = 334) retrieved from multivariable linear model.

	*B^a^*	95% CI	*P* ^b^
Intercept	−0.31		
BMI*^c^*			
Normal weight	Ref.		
Underweight	0.36	−0.20 to 0.91	0.204
Overweight	−0.10	−0.36 to 0.16	0.442
Obese	−0.56	−0.97 to −0.16	**0.007**
Cumulative cisplatin (100 mg·m^−2^)	−0.12	−0.21 to −0.02	**0.034**
Cumulative cranial RT (10 Gy)	−0.10	−0.19 to −0.01	**0.028**
Cumulative heart-relevant RT (10 Gy)^*d*,*e*^	−0.05	−0.14 to 0.04	0.325

Statistically significant associations are in bold. *^a^* The unstandardized regression coefficient indicates the change in the standard deviation of the 1-min STS *z*-score for BMI (underweight/overweight/obese vs normal weight) and for a one-unit change of cumulative doses of cyclophosphamide, cisplatin, cranial RT, and heart-relevant RT. *^b^ P* value retrieved from Wald test. *^c^* BMI was classified as underweight (<18.5 kg·m^−2^), normal weight (≥18.5–24.9 kg·m^−2^), overweight (25–29.9 kg·m^−2^), and obese (≥30 kg·m^−2^) (29). *^d^* According to the COG Guidelines Version 5.0 (i. e., chest, abdomen, whole or thoracic spine, TBI) (28). *^e^* Including survivors who received TBI. *B*, unstandardized regression coefficient; RT, radiotherapy.

### Change of Performance Over Time in 1-Min STS Test among ASCC with Increased Cardiovascular Risk

**FIGURE 3:**
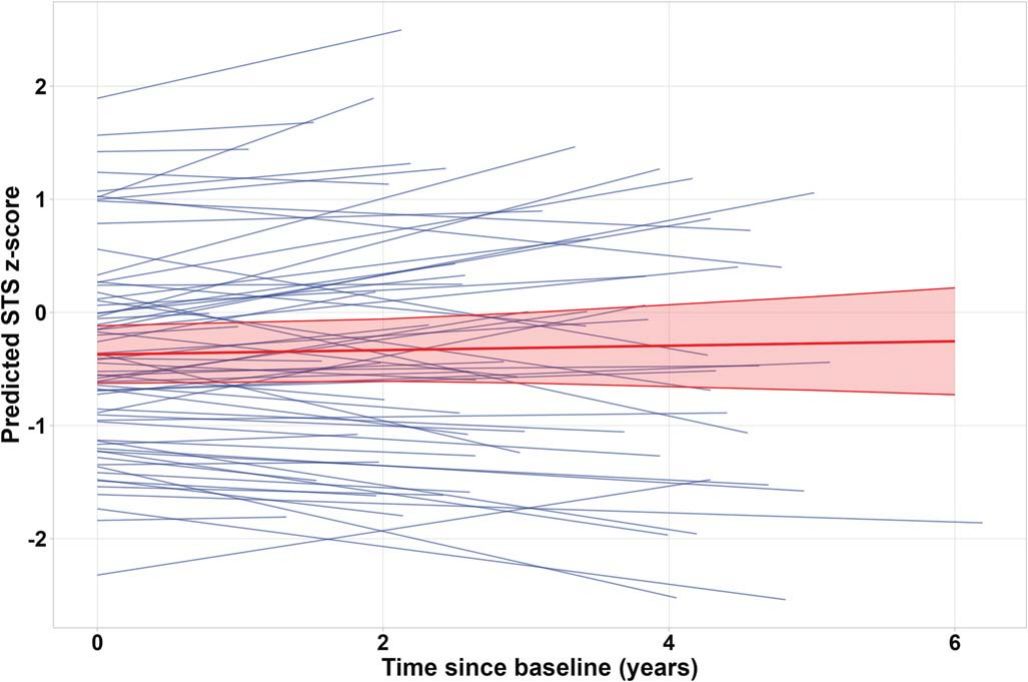
Spaghetti plot of 1-min STS *z*-scores in *N* = 67 ASCC who received cardiotoxic treatment with either anthracyclines and/or heart-relevant radiotherapy and who attended 1–3 follow-up visits after baseline examination.

For the longitudinal analysis of 1-min STS *z*-scores, we included 67 (15% of 441 CardioOnco study participants) ASCC at increased risk for cardiovascular disease. We present subgroup characteristics in Supplemental Tables 4A and 4B (Supplemental Digital Content, http://links.lww.com/MSS/D120). The number of participant follow-up visits ranged from 1 to 3. Median time between follow-up visits was 2.6 yr (IQR, 2.0–4.0 yr). We found no change in 1-min STS performance over the yr (fixed effects: *B* = 0.02; 95% CI, −0.05 to 0.09; see also Fig. 3). However, random effects of time (*B* = 0.05; 95% CI, 0.02–0.1) revealed heterogeneity over time, which means some participants improved, some participants deteriorated, and some participants remained stable in their 1-min STS performances over time.

## DISCUSSION

We found that ASCC had reduced lower body muscular strength and endurance when compared with the general population. We identified obesity, cisplatin chemotherapy, and CRT to be associated with a reduced STS performance. Lower body muscular strength and endurance did not change over 2–5 yr among ASCC at increased risk for cardiovascular disease.

Our results align with previous studies reporting deficits of lower body muscular strength and endurance among ASCC (3,21). In a study of 78 survivors of brain tumors with a median age of 22 yr at study, 55% of survivors versus 12% of controls showed diminished knee extension strength measured by handheld dynamometry (3). In a study of 365 survivors of ALL with a median age of 29 yr, survivors showed significantly decreased quadriceps strength and endurance compared with controls measured by isokinetic dynamometry (21). Our data showed obesity associated with diminished lower body muscular strength and endurance—a finding described before for both survivors of childhood ALL (8) and the general population (23). We further observed reduced 1-min STS performance among ASCC who received cisplatin treatment. ASCC treated with cisplatin can suffer from peripheral neuropathy up to 20 yr after treatment (38,39), causing numbness, loss of balance, and muscle weakness (40). Because STS performance depends on balance (15), peripheral neuropathy possibly explains some of our findings. We also found higher CRT dose associated with lower body strength and endurance. Evidence on this association is conflicting. One previous study showed CRT to be associated with a decreased knee extension strength among 365 adult survivors of ALL (21), whereas a second study by the same author found no association of CRT with impaired knee extension strength among 125 adult survivors of ALL (19). Of note, both studies only analyzed CRT as a categorical (yes/no) variable and did not study the dose–response relationship as in our case. Hormonal dysregulation after CRT can lead to reduced lean mass of ASCC (2) and thus decrease lower body strength and endurance. CRT damages white and gray matter of the brain leading to neurocognitive deficits (41), which possibly also affects 1-min STS test performance. We did not confirm (see Supplemental Table 3B, Supplemental Digital Content, http://links.lww.com/MSS/D120) previous studies reporting that asparaginase (4), vincristine (5), and hematopoietic stem cell transplantation (7,8) affected lower body muscular strength and endurance. However, risk factors identified by this study are probably not exhaustive. Muscle weakness, which would affect STS performance, is likely to arise from a combination of factors, including the cancer itself, cancer treatments, and systemic alterations such as inflammation, hormone levels, and nutritional status (2).

Lower body strength and endurance did not change over time among ASCC who received cardiotoxic treatment. To our knowledge, no study so far studied the course of lower body muscle strength among ASCC longitudinally. In a study with 18 children during and shortly after cancer treatment, individuals showed decreased knee extension and foot dorsal extension strength 6 months after the end of treatment (42). Our random effects analysis showed that 1-min STS performance trajectories varied among ASCC, with some improving and others declining over time. However, because of our small sample size, we could not investigate underlying reasons for different trajectories.

### One-Minute STS Test as a Novel Approach and Practical Implications

Our study uses the 1-min STS test in a multicenter setting to assess ASCC within routine clinical care. The 1-min STS test is easily and quickly performed in any clinical setting with basic equipment: a stopwatch and a standard armless chair (23). Although previous studies used mostly isokinetic or handheld dynamometry to assess lower body strength, and a broad variety of endurance tests (32), our study combines both aspects of physical fitness in one test. Implementing practical screening measures—such as the 1-min STS test—in clinical routine is valuable for identifying survivors at risk for such deficits (32). To prevent and treat muscle strength impairments, early targeted and individual clinical exercise therapy is necessary (32). For example, the German Network ActiveOncoKids implements physical activity and exercise within usual care for pediatric and adolescent patients during and after cancer treatment (43). Interventions focusing on improving lower body muscular strength and endurance among ASCC are scarce and showed limited effectiveness (44), demonstrating the need for future research.

### Strengths and Limitations

Our study—one of the largest using 1-min STS test among ASCC—extends understandings of lower body muscular strength and endurance. We used population-based Swiss reference values for comparison and included survivors with various cancer diagnoses. Not restricting our study population to survivors of one cancer diagnosis made our findings more generalizable. Including multiple study centers also allowed to have a larger sample size. However, study limitations should be considered. Because our study was multicentric, multiple assessors evaluated the 1-min STS test, which could have led to interobserver bias. However, as described previously, we took several measures to harmonize data collection across centers. Unfortunately, we were not able to ensure that weight and height were measured with the same device across centers, which could have introduced measurement bias. Our sensitivity analysis of weight measurements showed significant differences among centers, although there were no differences in height measurements. In some cases, diagnosis of dyslipidemia, hypertension, and diabetes mellitus was self-reported, which could have biased our findings because it is not known according to which international guidelines these diagnoses were made. Survivors had to attend the outpatient clinics to perform 1-min STS test, which may have been a source of selection bias because of a considerable effort that survivors needed to take. To evaluate the selection bias in our study, we previously compared participants with nonparticipants and showed that nonparticipants were younger and less often exposed to chemotherapy or hematopoietic stem cell transplantation (45). Given the fact that we calculated 1-min STS *z*-scores stratified by age groups, we think that age of participants did not substantially affect our findings. Because we excluded ASCC treated with only surgery and/or radiotherapy other than heart-relevant radiotherapy, our study design possibly introduced other selection bias; thus, survivors of low-grade central nervous system tumors, retinoblastomas, or germ cell tumors without systemic treatment are possibly underrepresented. Compared with other lower body muscular strength tests, the 1-min STS test requires some balance skills to be correctly performed (15). This needs to be considered when comparing our findings to other tests. Furthermore, levels of physical activity—an important predictor of muscle strength (46)—were not assessed in this study, which could have explained why lower body muscle strength of some ASCC changes over time. Our small sample size for the longitudinal analysis was likely underpowered to detect small changes over time and requires further investigation in studies with larger samples or more measurement time points (47). Finally, the observational design of the investigation of risk factors disallowed causal conclusions.

## CONCLUSIONS

Using a simple and practical screening tool—the 1-min STS test—we found evidence of reduced lower body muscular strength and endurance after treatment for childhood cancer, especially among survivors with obesity or treated with cisplatin or cranial radiation. Our findings suggest a need for effective training and rehabilitation programs for maintaining daily functioning and improving cardiovascular health and quality of life for survivors.
